# Transcriptional and post-transcriptional regulation of RNAi-related gene expression during plant-virus interactions

**DOI:** 10.1007/s44154-022-00057-y

**Published:** 2022-08-19

**Authors:** Qian Gong, Yunjing Wang, Zhenhui Jin, Yiguo Hong, Yule Liu

**Affiliations:** 1 MOE Key Laboratory of Bioinformatics and Center for Plant Biology, School of Life Sciences, Tsinghua University, Beijing, 100084 China; 2 Tsinghua-Peking Center for Life Sciences, Beijing, 100084 China; 3 Research Centre for Plant RNA Signaling, College of Life and Environmental Sciences, Hangzhou Normal University, Hangzhou, 311121 China; 4 School of Science and the Environment, University of Worcester, Worcester, WR2 6AJ UK; 5 School of Life Sciences, University of Warwick, Coventry, CV4 7AL UK

**Keywords:** Transcriptional regulation, RNAi, Gene expression, Virus, Plant immunity

## Abstract

As sessile organisms, plants encounter diverse invasions from pathogens including viruses. To survive and thrive, plants have evolved multilayered defense mechanisms to combat virus infection. RNAi, also known as RNA silencing, is an across-kingdom innate immunity and gene regulatory machinery. Molecular framework and crucial roles of RNAi in antiviral defense have been well-characterized. However, it is largely unknown that how RNAi is transcriptionally regulated to initiate, maintain and enhance cellular silencing under normal or stress conditions. Recently, insights into the transcriptional and post-transcriptional regulation of RNAi-related genes in different physiological processes have been emerging. In this review, we integrate these new findings to provide updated views on how plants modulate RNAi machinery at the (post-) transcriptional level to respond to virus infection.

## Introduction

Plants are persistently challenged by various phytopathogens. Among them, viruses, as obligatory intracellular parasites, can cause severe diseases and viral epidemics on all major crops of agronomic importance. To protect themselves, plants have evolved multilayered defense mechanisms against viruses including physical barriers, innate immunity, RNAi, and autophagy (Haxim et al., 2017; Ismayil et al., 2020; Lopez-Gomollon & Baulcombe, 2022; Soosaar et al., 2005).

**Fig. 1:**
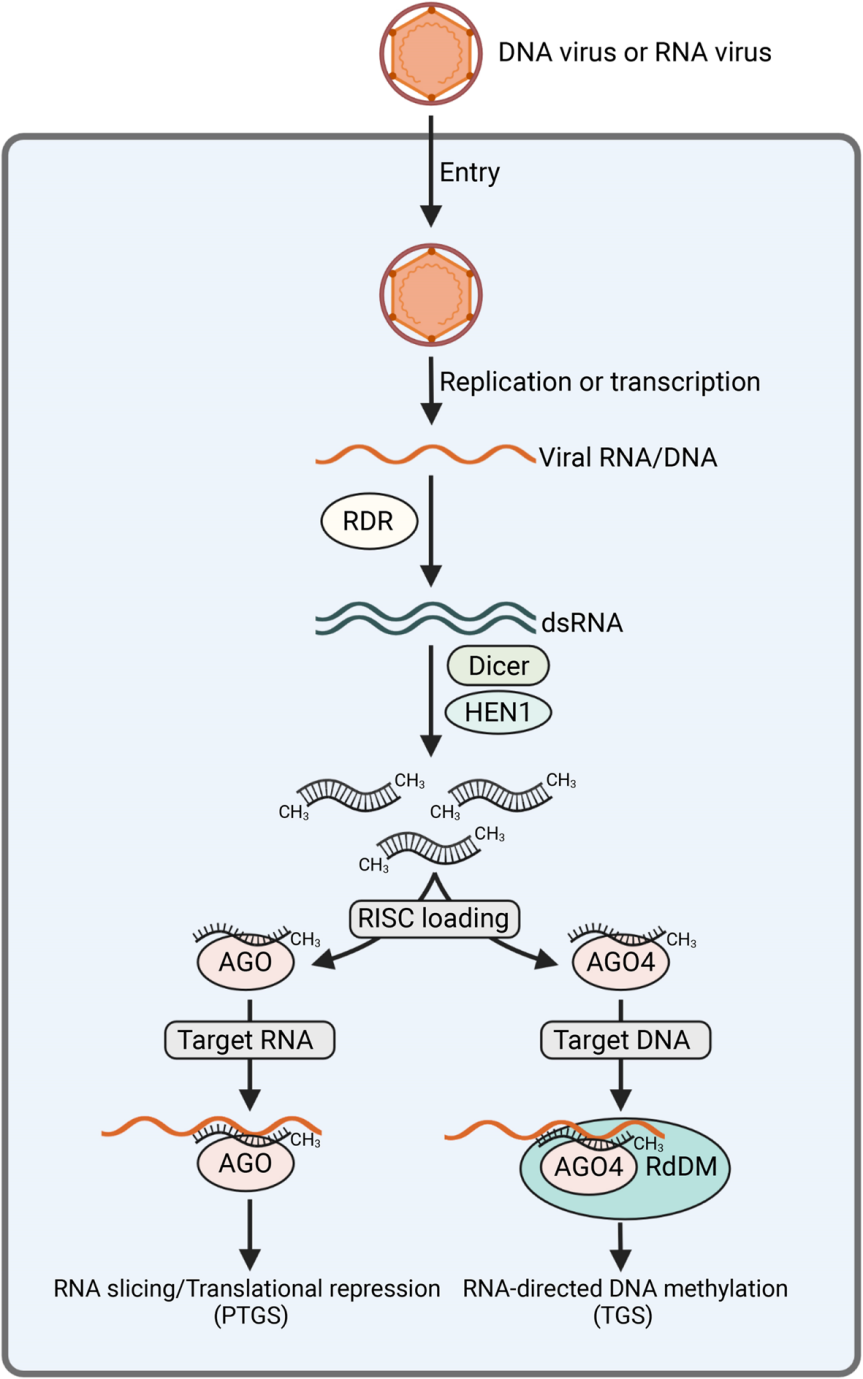
Schematic diagram depicting plant RNAi pathway activated by virus infection. Replication of RNA viruses produce dsRNA intermediates, and they serve as inducers of post-transcriptional gene silencing (PTGS). Infection by DNA viruses also induces RNA-directed DNA methylation (RdDM), which is involved in modification of DNA and/or histones, result in transcriptional gene silencing (TGS)

RNAi has been well-established as a significant mechanism to regulate development, genome stability, stress-induced responses, and basal defense against virus invasion (Baulcombe, 2004; Ding, 2010; Li & Wang, 2019). Plant viruses activate RNAi through double-stranded RNA (dsRNA) and viral small-interfering RNAs (vsiRNAs). These dsRNAs come from virus replication (for RNA viruses), de novo synthesized dsRNAs, intramolecular dsRNA structure, and bidirectional transcription of the viral genome (for DNA viruses) (Boualem et al., 2016; Guo et al., 2019; Matzke & Mosher, 2014). Virus-induced RNA silencing occurs in three steps: initiation, amplification, and spreading (Llave, 2010). Silencing is initiated when viral dsRNAs are recognized by Dicer-like (DCL) ribonucleases to generate 21 to 24 nt primary vsiRNAs. Amplification involves both RNA-dependent RNA polymerases (RDRs) and DCLs. RDRs use viral single-stranded RNAs (ssRNAs) as the template to synthesize long, perfect dsRNAs, which further serve as substrates for the DCL-dependent formation of secondary vsiRNAs (Garcia-Ruiz et al., 2010; Wang et al., 2011, 2010). Amplified vsiRNAs are able to spread throughout the plant and support the systemic silencing (Liu & Chen, 2018; Palauqui & Balzergue, 1999; Voinnet et al., 1998). Subsequently, vsiRNAs are loaded into distinct ARGONAUTE (AGO)-containing effector complexes to form RNA induced silencing complex (RISC), where they provide specificity for RNA or DNA targeting through a sequence homology-dependent mechanism (Peters & Meister, 2007; Vaucheret, 2008). The association of RISC with complementary target RNAs leads to cleavage, degradation, or translational inhibition of the cognate viral RNAs (Fang & Qi, 2016; Garcia-Ruiz et al., 2015; Jaubert et al., 2011; Wu et al., 2015; Zhang et al., 2006), while the interaction with target viral DNA causes modification of DNA and/or histones, result in transcriptional repression (Raja et al., 2008) (Fig. 1). Although the functions of genes encoding proteins involved in RNA silencing were well-characterized in plants, the regulatory mechanism of their transcription remains elusive. In this review, we will highlight recent advances on transcriptional and post-transcriptional regulation of RNAi-related gene expression and discuss how miRNAs, phytohormones, and viral pathogens influence RNAi-related gene expression during the plant-virus warfare.

## Regulation of miRNAs and their roles in plant-virus interactions

Most plants possess a large number of microRNA (*MIR*) genes, mainly in intergenic regions throughout the genome (Yu et al., 2017). Most *MIR* genes possess their own transcriptional unit. *MIR* genes are transcribed into pri-miRNAs by RNA polymerase II (Pol II) (Stepien et al., 2017; Xie et al., 2005), and pri-miRNAs are ultimately processed into small (size of 20–24 nucleotides long) ssRNAs, termed as miRNAs (Liu et al., 2012; Song et al., 2010; Zhu et al., 2013). The mature miRNAs guide strand can be loaded into an AGO protein to form RISC. The PIWI domain of AGO proteins forms an RNase H-like fold with a slicer endonuclease activity, which is capable of cleaving target RNAs that are complementary to the loaded guide strand (Song et al., 2004). MiRNAs play key roles in the regulation of their targeted genes expression in plants.

In plant-virus interactions, miRNAs were used as weapons for both host and pathogen. MiRNAs play important roles in antiviral immunity by targeting endogenous genes, including RNA silencing components, hormone signaling pathways, and nucleotide binding site-leucine-rich repeat (NBS-LRR) resistance (*R*) genes (Table 1) (Jin et al., 2021; Mlotshwa et al., 2008; Zhang et al., 2016). For example, upon *Rice stripe virus* (RSV) infection, miR528 becomes preferentially associated with AGO18, leading to increased L-ascorbate oxidase (AO) activity, increased basal active oxygen accumulation and enhanced antiviral defense in rice (Wu et al., 2017). RSV infection also induce miR444 transcription and diminishes the repressive effects of the MADS box genes on *RDR1* transcription, thus activating RDR1 dependent antiviral silencing pathway (Wang et al., 2016). In terms of miRNAs regulation of *R* genes, bra-miR1885 which targets TIR-NBS-LRR class *R* gene is specifically induced by TuMV infection in brassica (He et al., 2008). MiR482/2118 family is found to target NB-LRR encoding genes in tomato, while nta-miR6019 and nta-miR6020 guide cleavage of transcripts of tobacco NB-LRR immune receptor N that confers resistance to *Tobacco mosaic virus* (TMV) (Li et al., 2012a; Shivaprasad et al., 2012; Zhai et al., 2011). In addition, RNAi can be controlled by miRNAs` feedback regulation of RNAi related genes expression. For example, miR162, miR168, and miR403 targets *DCL1*, *AGO1*, and *AGO2/3* mRNA, respectively (Allen et al., 2005; Vaucheret et al., 2004; Xie et al., 2003).

**Table 1:** List of miRNAs involed in plant-virus interactions

miRNAs	Targets	Hosts	Pathogen	References
miR159	MYB33/55	Arabidopsis	*Cucumber mosaic virus*	(Du et al., 2014)
miR162	DCL1	Arabidopsis	*Cucumber mosaic virus*	(Zhang et al., 2006)
			*Turnip yellow mosaic virus*	(Xie et al., 2003)
miR168	AGO1	Arabidopsis	*Turnip crinkle virus*	(Varallyay et al., 2010)
miR1885	TIR-NBS-LRR gene	*Brassica rapa*	*Turnip mosaic virus*	(He et al., 2008)
miR162	DCL2	*Gossypium hirsutum*	*Cotton leafroll dwarf polerovirus*	(Silva et al., 2011)
miR168/miR395ad	C1, C3, C4, V1, V2	*Gossypium hirsutum*	*Cotton leaf curl Burewala virus*	(Shweta et al., 2018)
miR398	C1, C4, V1, IR	*Gossypium hirsutum*	*Cotton leaf curl Multan virus*	(Akmal et al., 2017)
miR398	umecyanin	*Nicotiana benthamiana*	*Beet necrotic yellow vein virus*	(Liu et al., 2020)
miR6019	Receptor N	*Nicotiana tabacum*	*Tobacco mosaic virus*	(Li et al., 2012a)
miR6020	Receptor N	*Nicotiana tabacum*	*Tobacco mosaic virus*	(Li et al., 2012a)
miR164	NAC	*Oryza sativa*	*Rice ragged stunt virus*	(Zhang et al., 2016)
miR168	AGO1a	*Oryza sativa*	*Rice stripe virus*	(Wu et al., 2015)
miR171b	SCL6-IIa/b/c	*Oryza sativa*	*Rice stripe virus*	(Tong et al., 2017)
miR319	TCP genes	*Oryza sativa*	*Rice ragged stunt virus*	(Zhang et al., 2016)
miR444	MADS23/27a/57	*Oryza sativa*	*Rice stripe virus*	(Wang et al., 2016)
miR528	AO	*Oryza sativa*	*Rice stripe virus*	(Wu et al., 2017)
miR396	vital ORF3	*Saccharum officinarum L*	*Sugarcane Bacilliform Guadeloupe A Virus*	(Ashraf et al., 2020)
miR164	NMO	*Triticum aestivum*	*Rice black streaked dwarf virus*	(Zhang et al., 2016)
miR319	PCF8	*Triticum aestivum*	*Rice black streaked dwarf virus*	(Zhang et al., 2016)

Viral infection can alter the pattern of miRNA expression in plants. RNA viruses including *Cucumber mosaic virus* (CMV; Cucumovirus) (Feng et al., 2014), *Turnip mosaic virus* (TuMV; Potyvirus) (Wang et al., 2015), *Potato virus X* (PVX; Potexvirus) (Pacheco et al., 2012), *Cucumber green mottle mosaic virus* (CGMMV; Tobamovirus) (Liu et al., 2015), *Oilseed rape mosaic tobamovirus* (ORMV; Tobamovirus) (Hu et al., 2011), *Rice black-streaked dwarf virus* (RBSDV; Fijivirus) (Sun et al., 2015; Xu et al., 2014), *Hibiscus chlorotic ringspot virus* (HCRSV; Carmovirus) (Gao et al., 2013), and the DNA virus such as *Tomato leaf curl virus* (ToLCV; Begomovirus) (Naqvi et al., 2010), have all been reported to affect host miRNA expression. For example, tobamoviruses or potyviruses infection alter the accumulation of miRNAs such as miR156, 160, 164, and 171 in *Nicotiana tabacum* (Bazzini et al., 2007). PVX and either *Potato virus Y* (PVY) or *plum pox virus* (PPV) co-infection causes more miR156, 171, 398, and 168 accumulation than single infections in *Nicotiana benthamiana* (Pacheco et al., 2012). MiR168a, miR403a, miR162b, and miR1515a are upregulated during *Soybean mosaic virus* (SMV) infection. Viral symptoms including chlorosis, necrosis, curling, and stunting are often associated with alterations of miRNAs (Pelaez & Sanchez, 2013). For instance, disease symptom development caused by *Rice ragged stunt virus* (RRSV) infection is associated with the induction of miR319, and the reduced accumulation of rice miR171b in RSV-infected plants contributes to RSV specific disease symptoms (Tong et al., 2017; Zhang et al., 2016). Leaf curl symptom caused by *Tomato leaf curl new Delhi virus* (ToLCNDV) infection is associated with induction of miR159/319 and miR172 in tomato, and development abnormalities or viral symptoms caused by TMV Cg or ORMV infection in Arabidopsis are associated with induction of miR164a (Bazzini et al., 2009; Naqvi et al., 2010).

Although alternation of miRNA expression or activity during viral infection has been found extensively, the regulation mechanism for these cases is largely unknown. Both plant and viral protein can cause differential miRNA expression and activity. For examples, rice SQUAMOSA Promoter Binding Protein-Like 9 (SPL9) binds to* miR528* promoter and activates *miR528* gene expression as the transcription factor (TF) in rice plants (Yao et al., 2019). In addition, RSV infection enhances jasmonic acid (JA) biosynthesis and signaling of the infected plants, leading to the release of JA-induced TF JAMYB. JAMYB binds to and activates the *AGO18* promoter. AGO18 is found recruiting a large amount of miR168 through small RNA deep sequencing analyses of purified AGO18-containing complexes, further relieves the repression of miR168 on *AGO1* mRNA in RSV-infected rice (Wu et al., 2015; Yang et al., 2020). As a major effector of antiviral RNA silencing, AGO1 associates with vsiRNAs and mediates degradation of viral RNAs (Wu et al., 2015; Yang et al., 2020). Little is known about how plants sense initial cues to mobilize RNAi. Recently, we found that mechanical wounding or aphid feeding to *Nicotiana benthamiana* cells during virus intrusion activates calmodulin-binding transcription activator-3 (CAMTA3) function, which directly binds to *Bifunctional nuclease-2* (*BN2*) and *RDR6* promoters and induces their transcription. BN2 stabilizes *AGO1/2* and *DCL1* mRNA levels by degrading their cognate microRNAs (Wang et al., 2021; 2022). Therefore, multiple RNAi components are primed for combating virus invasion. Viruses also took full advantage of miRNAs for the effective infection. RSV NS3 (P3) Protein suppresses RNA silencing to regulate the expression of multiple host resistance-associated miRNAs upon RSV infection (Shen et al., 2010; Zheng et al., 2017). Besides, many viruses encode viral suppressor of RNA silencing (VSR) such as P19 from Tombusvirus and P1/HC-Pro from TuMV to enhance virus infection by regulating host miRNAs biogenesis, activity, or accumulation (Liu et al., 2020; Zhang et al., 2006).

## Regulation of ta-siRNAs and their roles in plant-virus interactions

Another class of endogenous sRNAs which have important roles during plant-virus interactions are trans-acting siRNA (ta-siRNA). Ta-siRNA is a subset of phasiRNAs encoded by *TAS* genes that can regulate target genes via mRNA cleavage in trans (Allen et al., 2005; Fei et al., 2013; Yoshikawa et al., 2005). The biogenesis of ta-siRNAs is initiated by miRNA-mediated cleavage of *TAS* transcripts. The cleaved RNAs are copied into dsRNAs by RDR6, and dsRNAs are cleaved to generate multiple ta-siRNAs by type III ribonuclease in a phased manner (Axtell et al., 2006). To date, four families of *TAS* genes with eight loci have been discovered in the Arabidopsis genome: *TAS1*, *TAS2*, *TAS3*, and *TAS4* (Chen, 2009). TAS1 and TAS2 require miR173 for ta-siRNA biogenesis, whereas TAS3 and TAS4 require miR390 and miR828, respectively (Allen et al., 2005; Peragine et al., 2004; Rajagopalan et al., 2006; Vazquez et al., 2004; Yoshikawa et al., 2005). TAS1 and TAS2 only exist in certain plant species, however, TAS3 and TAS4 are conserved (Allen & Howell, 2010; Xia et al., 2017).

Ta-siRNAs are involved in plant-virus interactions and induced during the infection of plant with pathogens including ToLCNDV (Singh et al., 2015). In addition, ta-siRNAs are generated and transported systemically within 4 to 6 h of primary pathogen infection to induce systemic acquired resistance (SAR) (Shine et al., 2022). Viruses also employ different strategies to suppress ta-siRNAs generation. For instance, a small peptide VISP1 is reported to compromise antiviral immunity by inducing autophagic degradation of SGS3 to inhibit SGS3/RDR6-dependent viral siRNA amplification and endogenous ta-siRNAs biogenesis during CMV infection (Tong et al., 2021). CMV 2b protein also interferes with the production of ta-siRNAs through interaction with AGO1 (Feng et al., 2013). Apart from CMV, transactivator/viroplasmin (TAV) protein of *Cauliflower mosaic virus* (CaMV), p2 protein of RSV, TGBp1 of *Plantago asiatica mosaic virus* (PlAMV), and coat protein (CP) of HCRSV are capable of interfering with ta-siRNAs biogenesis, mainly via interaction with SGS3/RDR6 bodies (Du et al., 2011b; Meng et al., 2008; Okano et al., 2014; Shivaprasad et al., 2008). In addition, syn-tasiRNAs can be designed to target virus in plants. Syn-tasiRNA contains a functional *TAS* precursor in which a subset of the endogenous ta-siRNA sequences is substituted by one or several designed syn-tasiRNA sequences in tandem (Chen et al., 2016; Cisneros & Carbonell, 2020; Miao et al., 2021). Indeed, syn-tasiRNAs can confer virus resistance in multiple plant species (Carbonell & Daros, 2017; Carbonell et al., 2019a, 2019b).

## Effects of phytohormone on the expression of RNAi components

**Fig. 2:**
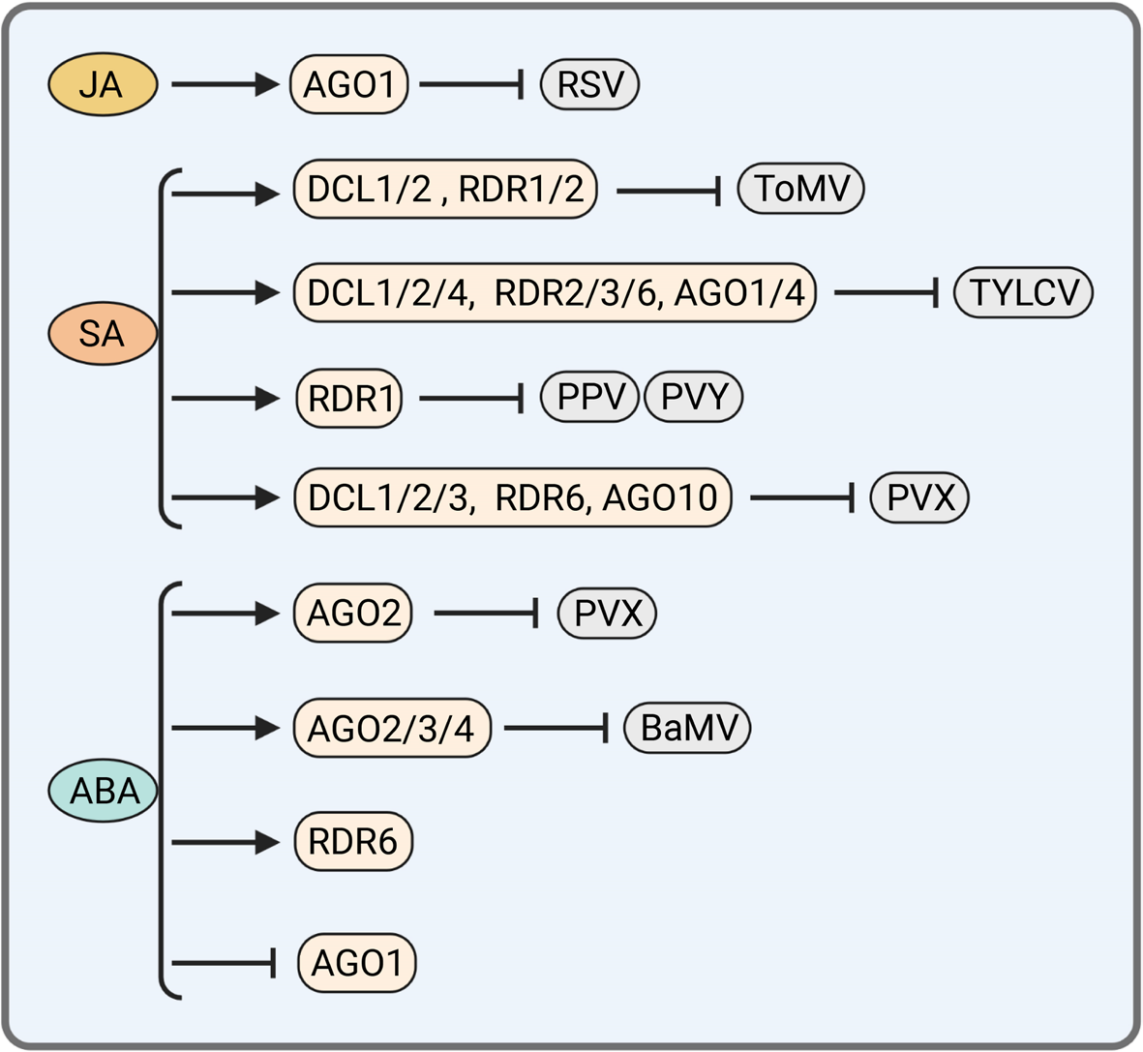
The roles of plant hormones in RNAi-related gene expression during virus infection. The plant hormones shown in ovals generally have positive or negative effects on different RNAi-related gene expression. Correlation among hormones, RNAi-related key genes, and viruses are shown. Arrow and T-sign indicate positive or negative impact, respectively

Phytohormones are required for plant development and response to biotic or abiotic stresses. Numerous findings have revealed the significance of not only individual phytohormones or separate signaling cascades but also complex network of intersecting hormone signal pathways in antiviral immunity (Alazem & Lin, 2015; Collum & Culver, 2016). However, the cross-talk between phytohormones and RNAi is very complicated, and more attention is needed to understand the effects of hormones on the regulation of antiviral RNAi (Fig. 2).

JA is a key regulator of defense responses to necrotrophic pathogens as well as insect infestation in plants (Chini et al., 2016; Wasternack, 2014; Yan et al., 2018; Zhang et al., 2017). JA is also involved in plant antiviral defense (Jia et al., 2016; Yang et al., 2020). However, the connection between JA and RNAi pathway has not been clear. A recent report has linked JA to the expression of an RNAi component during virus infection. In this study, RSV CP triggers JA biosynthesis and signaling, leading to the degradation of JAZ proteins and the release of TF JAMYB. JAMYB binds to and activates *AGO18* promoter to enhance the transcription of *AGO18*, which further increases rice antiviral RNAi defense by sequestering miR168 and releasing *AGO1* mRNA (Wu et al., 2015; Yang et al., 2020) (Fig. 2).

Salicylic acid (SA) plays a vital role in plant immunity including antiviral defense (Yan & Dong, 2014). SA treatment significantly induces transcription of *DCL1/2, RDR1/2* in tomato, leading to a repression of *Tomato mosaic virus* (ToMV) infection (Campos et al., 2014). Similarly, exogenous SA application significantly triggers the transcription of RNAi pathway genes including *DCL1/2/4*, *RDR2/3a*, *RDR6a*, and *AGO1/4* in tomato, enhances the resistance to *Tomato yellow leaf curl virus* (TYLCV) (Li et al., 2018). Besides, SA is able to induce *RDR1* expression in tobacco and Arabidopsis to defense PPV, PVY, and other viruses by enhancing vsiRNA biogenesis (Alamillo et al., 2006; Hunter et al., 2013; Lee et al., 2016; Rakhshandehroo et al., 2017). The biocontrol agent ZhiNengCong (ZNC), which is the extraction of an endophytic fungus, increases SA content along with positive regulation of *DCL3*, *AGO10*, and other RNAi-related gene expression to enhance tobacco resistance against PVX in wild-type tobacco plants, but failed to induce those protective effects in transgenic *NahG* plants expressing SA-degrading enzyme salicylate hydroxylase (Peng et al., 2020). Interestingly, some SA-related TFs are co-expressed with *AGO*, *DCL*, and *RDR* genes, and the promoter regions of these *AGO*, *DCL*, and *RDR* genes arepredicted to contain the multiple binding sites for the corresponding SA-related TFs (Alazem et al., 2019) (Fig. 2). These results indicate that SA is able to modulate RNAi-related gene expression to repress virus infection.

SA and abscisic acid (ABA) are often antagonistic and regulate different stress responses, however, they have similar effects on antiviral immunity, which are partially achieved through RNAi pathway. The regulatory role of ABA in RNAi pathway isuncovered by Arabidopsis ABA deficient mutants *aba1-5* (Leon-Kloosterziel et al., 1996). In *aba1-5* plants, the expression level of *AGO1* is significantly increased, suggesting that ABA negatively regulates *AGO1* expression (Li et al., 2012b). Additionally, miR168a, a negative regulator of *AGO1*, is upregulated by ABA (Laubinger et al., 2010; Li et al., 2012b). Apart from that, impairment of the ABA pathway in *Arabidopsis thaliana* reduces the accumulation of *AGO2* and weakens resistance to PVX (Jaubert et al., 2011). ABA upregulates the expression of *AGO2*, *AGO3*, and *AGO4* to enhance resistance to *Bamboo mosaic virus* (BaMV) (Alazem et al., 2017). ABA also positively regulates *RDR6* gene expression and post-transcriptional gene silencing in rice cells (Yang et al., 2008) (Fig. 2). Notably, multiple RNA-silencing mutants, such as *dcl1*, *ago1*, *hen1*, *se-1*, and *hyl1* have ABA-hypersensitivity (Li et al., 2012b; Lu & Fedoroff, 2000; Zhang et al., 2008). These studies have allured more attention to the effects of hormones on transcriptional regulation of antiviral RNAi components. In view of the cross-talk between phytohormones and RNAi, there are still some crucial unsolved questions that need to be further characterized. For instance, 1) we need further investigation into how these hormones affect the key genes (*DCLs*, *RDRs*, and *AGO*s) in RNAi pathway. 2) Apart from *DCL*, *RDR*, and *AGO* family genes, are there any other genes which regulate or maintain the integrity of RNAi pathway modulated by these hormones? 3) Some genes are able to be transcriptionally regulated by multiple hormones with antagonism pattern, therefore, how hormones coordinate the regulation of RNAi-related gene expression? For instance, SA and ABA exhibited mutual antagonism of *AGO1* and *RDRs* expression (Alazem et al., 2019). ABA clearly induced expression of those genes only in the SA mutant *sid2-1*, however, both SA and ABA show similar regulation for other genes, for example, ABA-mediated *AGO2* induction is SA-dependent (Alazem et al., 2019). Besides, although the contribution of SA/JA signaling molecules in plant defense differs and depends on the type of invading pathogen, these two signaling pathways influence each other via a complex network of synergistic and antagonistic interactions (Alazem & Lin, 2015; Collum & Culver, 2016). The RNAi regulation by phytohormones are not simple linear or isolated cascades, but exhibit cross-talk with each other. Alteration in endogenous phytohormone levels seems to be a direct consequence of virus infection and is tightly coordinated with viral movement, replication, symptom development, and defense responses (Casteel et al., 2015; Collum et al., 2016; Tao et al., 2017; Zhao & Li, 2021). Hijacking host components in the phytohormone pathways is a common strategy in viral pathogenesis (Zhao & Li, 2021). Identifying the roles of phytohormones in viral infection and cross-talk with antiviral RNAi defense among different phytohormones pathways are challenges for the forthcoming years. We still lack specific molecular basis of phytohormones regulation of RNAi-related gene transcription. The comprehensive mechanism of signal integration among multiple phytohormones to regulate RNAi also needs further investigation.

## The effect of viral infection on RNAi-related gene expression

**Fig. 3:**
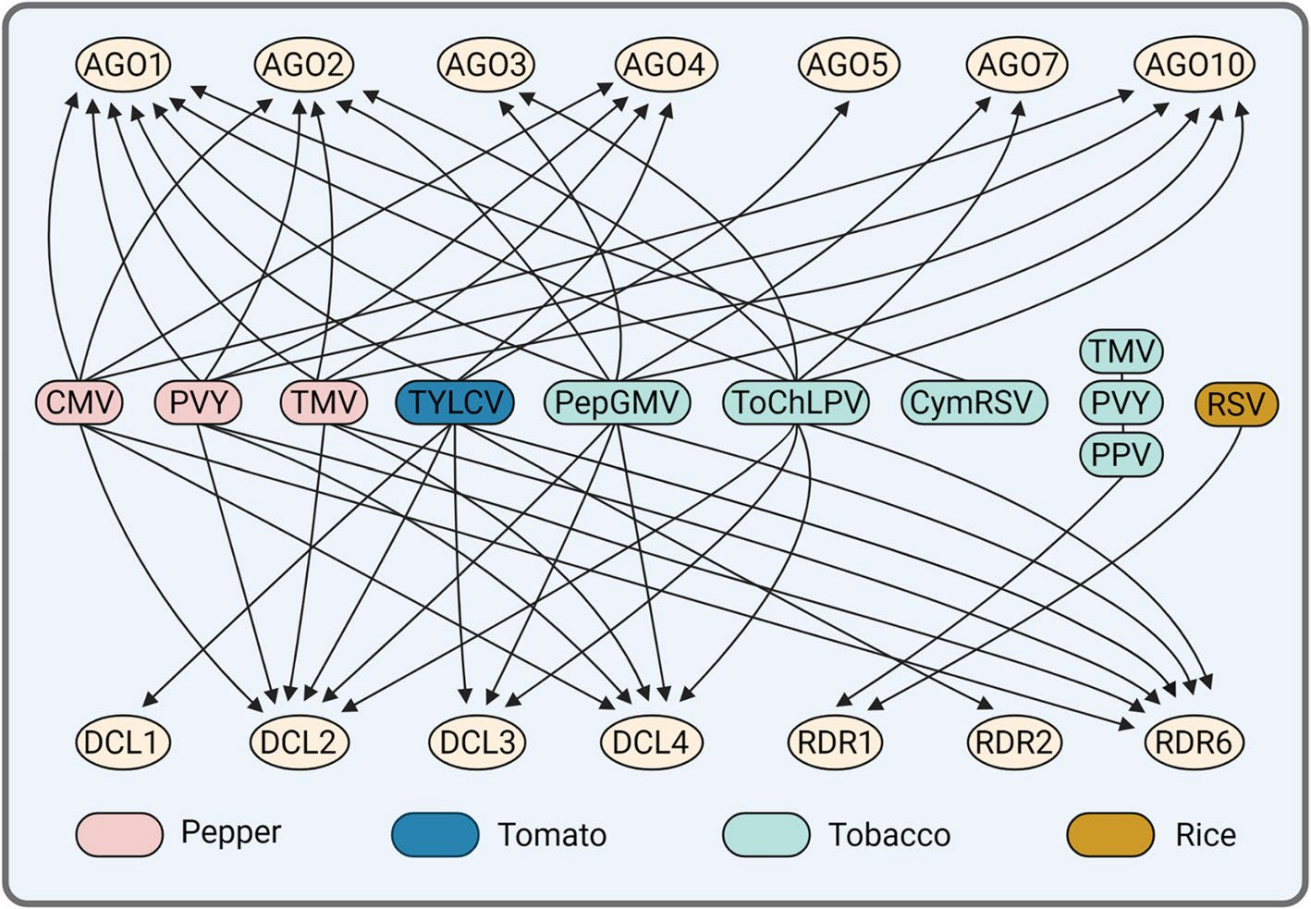
Different host and virus lead to diverse RNAi components expression. The RNAi components shown in ovals generally have been induced by different viruses

Viral infection often activates or up-regulates expression of host RNAi-related genes and this virus-resistant strategy seems more general in *Solanaceae* family plants (Fig. 3). For example, the transcription of multiple *AGOs* (*AGO1, AGO2, AGO4*, and *AGO10)*, *RDR6*, *DCL2*, and *DCL4* are upregulated with CMV, PVY, or TMV infection in pepper (Qin et al., 2018). Also, expression profiling of genes in TYLCV infected tomato showed that multiple RNAi core genes including *SlDCL1/2/3*, *SlRDR2*, *SlRDR6*, and five *AGO* genes (*SlAGO1a*, *1b*, *4a*, *4b*, and *5*) are triggered with high level expression in response to virus infection (Bai et al., 2012). In *Nicotiana benthamiana*, expression of repeat sequence fragments from both *Pepper golden mosaic virus* (PepGMV) and *Tomato chino La Paz virus* (ToChLPV) are able to upregulate *DCL2/3/4*, *AGO1/2/3*, *AGO7*, *AGO10*, and *RDR6* transcripts (Vargas-Salinas et al., 2021). *AGO1* mRNA level is elevated in *Cymbidium ringspot virus* (CymRSV) infected plants (Havelda et al., 2008). *RDR1* is also reported to be virus or SA inducible in different plants including Arabidopsis, *Nicotiana*, *Medicago truncatula*, maize (*Zea mays*), and rice (*Oryza sativa*) (Alamillo et al., 2006; Du et al., 2011a; He et al., 2010; Satoh et al., 2010; Yang et al., 2004). For instance, expression of the *Nicotiana tabacum RDR1* gene is induced by TMV, PVY, and PPV (Rakhshandehroo et al., 2009). In cucumber, four *RDR1* homologous genes are regulated with different expression profiles during virus infection. *RDR1b* is constitutively expressed at a high level only in resistant plants, whereas *RDR1c1* and *RDR1c2* are barely expressed in healthy plants, but induced to high levels by RNA and DNA virus infection (Kumari et al., 2021; Leibman et al., 2018). Besides, RSV infection induces miR444 accumulation, which enhances *OsRDR1* expression, leading to rice resistance to RSV infection (Wang et al., 2016). Although similar results have been repeatedly achieved, the regulation mechanism for those cases, especially the initial cue which provokes these responses is still elusive. Moreover, it also remains unclear how RNAi-related genes are transcriptionally and post-transcriptionally regulated. Recently, we revealed that a Ca^2+^-calmodulin-CAMTA3 cascade which may supply some missing part of the “puzzle”. We found that mechanical wounding or aphid feeding to *Nicotiana benthamiana* cells during virus intrusion activates RNAi-related gene expression through calcium signaling (Wang et al., 2021). A rapid wound-induced elevation in calcium fluxes triggers calmodulin-dependent activation of CAMTA3, which activates *RDR6* and *BN2* transcription. BN2 stabilizes mRNAs encoding key RNAi machinery components *AGO1/2* and *DCL1* by degrading their cognate microRNAs (Wang et al., 2021; 2022). Consequently, multiple RNAi-related genes expression is primed for combating virus invasion. These findings demonstrate that calcium signaling can act as a cue to up-regulate and tune the RNAi machinery.

**Fig. 4:**
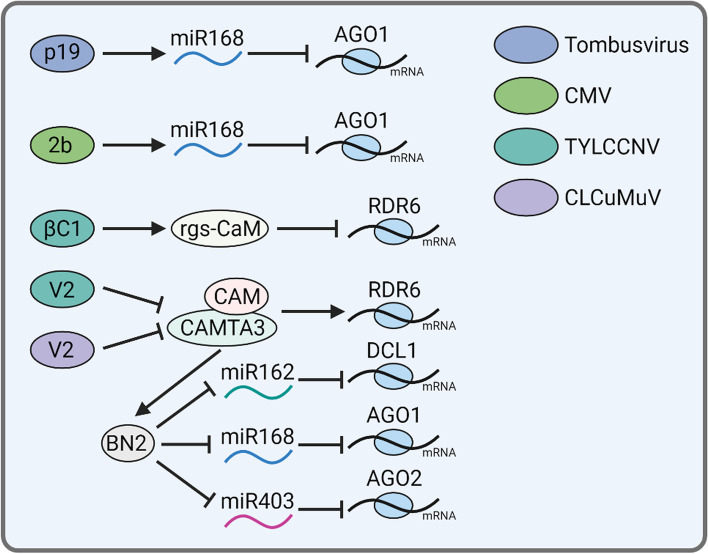
Viral protein regulates host RNAi-related gene expression during plant–virus interaction. Tombusvirus p19 mediates the induction of the miR168 expression to down-regulate endogenous *AGO1* mRNA level. CMV 2b protein inhibits miRNA pathways by blocking AGO1 cleavage activity to upregulate miR168. TYLCCNV-encoded VSR βC1 represses *RDR6* expression through transcript induction of an endogenous suppressors of RNAi, Nbrgs‐CaM protein to fight against the host RNA‐silencing‐mediated defense. CLCuMuV and TYLCCNV V2 proteins can disrupt the CaM-CAMTA3 interaction to impair CAMTA3-mediated transcriptional activation of both *RDR6* and *BN2*, result in suppression of antiviral RNAi. Arrow and T-sign indicate positive or negative impact, respectively

Furthermore, to survive, viruses encode proteins to counteract host RNAi-related gene activation as well (Fig. 4). For instance, tombusvirus infection enhances mRNA level of *AGO1* to resist virus infection, however, tombusvirus p19, as a RNA-silencing suppressor, mediates the induction of the miR168 expression to down-regulate endogenous *AGO1* mRNA level and inhibit the translational capacity of *AGO1* mRNA (Varallyay et al., 2010). Another example is the CMV encoded 2b suppressor protein. CMV 2b is found to inhibit miRNA pathways by blocking *AGO1* cleavage activity to upregulate miR168 and miR162 levels. Since miR162 and 168 negatively regulate the RNAi by targeting *DCL1* and *AGO1* mRNAs, respectively, CMV 2b attenuates antiviral RNAi and counters host defense (Zhang et al., 2006). Similar observation is obtained from *Beet necrotic yellow vein virus* (BNYVV) infected plants. Characterization of the *Nicotiana benthamiana* miRNA profile in response to the BNYVV infection reveals that miR168 is induced during virus infection. Furthermore, up-regulated miR168 is also found in 22 other combinations of different plants and VSRs (Liu et al., 2020), indicating that the upregulation of miR168 commonly occurs during plant–virus interactions, and it is not related to the host species and the mode in which different VSRs act (Liu et al., 2020). Moreover, *Tomato yellow leaf curl China geminivirus* (TYLCCNV) encodes VSR βC1 to fight against the host RNAi‐mediated defense. βC1 induces a *calmodulin‐like* (*Nbrgs‐CaM*) gene expression, and Nbrgs‐CaM suppresses the production of secondary siRNAs, likely through repressing *RDR6* expression (Li et al., 2014). Another interesting strategy by which geminiviruses employ is uncovered recently. During virus invasion, the rapid wound-induced elevation in calcium fluxes triggers calmodulin-dependent activation of CAMTA3, which activates *RDR6* and *BN2* transcription. BN2 stabilizes *AGO1*/*2* and *DCL1* mRNAs, by degrading their cognate microRNAs. V2 proteins encoded by *Cotton Leaf Curl Multan virus* (CLCuMuV) and TYLCCNV can disrupt the calmodulin-CAMTA3 interaction, which further impair CAMTA3-mediated transcriptional activation of both *RDR6* and *BN2* to suppress antiviral RNAi (Wang et al., 2021; 2022).

## Conclusions and perspectives

Over the past decades, RNAi has become a research hotspot in the research field of plant-virus interactions. RNAi plays a significant role in regulating defense against virus invasion by degrading RNA or modifying DNA through siRNAs. However, how RNAi is transcriptionally regulated to initiate, maintain, and enhance cellular RNAi machinery during virus infections still await to be uncovered. Current studies are extending knowledge concerning the correlation between RNAi and different physiological factors such as phytohormone or pathogens, however, more information is required for elucidating the mechanism of fine-tuning RNAi machinery on RNAi-related gene transcriptional control. To unveil the truth, we need to confront following challenges in forthcoming years. 1) New genes or small RNAs associated with antiviral RNAi need to be identified. 2) How RNAi key genes are transcriptionally regulated by phytohormones or virus infection still awaits to be discovered. 3) Since transcriptional regulation of RNAi machinery is associated with multiple aspects, challenge lies in deciphering emerging picture of complex mechanisms which are not simple linear or isolated cascades, but exhibit cross-talk. Furthermore, challenge still lies in translating the basic knowledge gained from model species to crops. In summary, addressing how RNAi is transcriptionally and post-transcriptionally regulated in plant-virus interactions will advance our understanding of RNAi machinery and elucidate how plant recognizes different stress and responses through RNAi. Future research in this field will surely yield more exciting discoveries and support development of plant antiviral immunity.

## Data Availability

Not applicable.

## Abbreviations

ABA: Abscisic acid
AGO: ARGONAUTE
AO: L-ascorbate oxidase
BaMV: *Bamboo* *mosaic* *virus*
BN2: *Bifunctional* *nuclease-2*
BNYVV: *Beet* *necrotic* *yellow vein virus*
Nbrgs‐CaM: Calmodulin‐like
CAMTA3: Calmodulin-binding transcription activator-3
CaMV: *Cauliflower* *mosaic* *virus*
CGMMV: *Cucumber* *green* *mottle* *mosaic* *virus*
CLCuMuV: *Cotton* *Leaf* *Curl* *Multan* *virus*
CMV: *Cucumber* *mosaic* *virus*
CP: Coat protein
CymRSV: *Cymbidium* *ringspot* *virus*
DCL: Dicer-like
HCRSV: *Hibiscus* *chlorotic* *ringspot* *virus*
JA: Jasmonic acid
JAMYB: JA-induced MYB
MIR: MicroRNA
ORMV: *Oilseed rape mosaic* *tobamovirus*
PepGMV: *Pepper golden mosaic virus*
Pol II: RNA polymerase II
PPV: *Plum* *pox* *virus*
PTGS: Post-transcriptional gene silencing
PlAMV: *Plantago* *asiatica* *mosaic* *virus*
PVX: *Potato* *X* *virus*
PVY: *Potato* *virus* *Y*
RBSDV: *Rice **black**-streaked dwarf* *virus*
RdDM: RNA-directed DNA methylation
RDRs: RNA-dependent RNA polymerases
RISC: RNA induced silencing complex
RRSV: *Rice* *ragged* *stunt* *virus*
RSV: *Rice* *stripe* *virus*
SA: Salicylic acid
SAR: Systemic acquired resistance
SMV: *Soybean* *mosaic* *virus*
SPL9: SQUAMOSA Promoter Binding Protein-Like 9
ssRNAs: Single-stranded RNAs
ta-siRNA: Trans-acting siRNA
TAV: Transactivator/viroplasmin
TF: Transcription factor
TGS: Transcriptional gene silencing
TMV: *Tobacco* *mosaic* *virus*
ToChLPV: *Tomato* *chino* *La* *Paz* *virus*
ToLCNDV: *Tomato* *leaf* *curl **new* *delhi* *virus*
ToLCV: *Tomato* *leaf* *curl* *virus*
ToMV: *Tomato mosaic* *virus*
TuMV: *Turnip* *mosaic* *virus*
TYLCCNV: *Tomato* *yellow* *leaf* *curl* *China* *geminivirus*
TYLCV: *Tomato* *yellow* *leaf* *curl* *virus*
vsiRNAs: Virus-derived short-interfering RNAs
VSR: Viral suppressor of RNA silencing
ZNC: ZhiNengCong
